# Exploring cultural imaginaries of robots with children with brittle bone disease: a participatory design study

**DOI:** 10.1136/medhum-2024-013039

**Published:** 2024-12-19

**Authors:** Christina E Stimson

**Affiliations:** 1 School of Computer Science, The University of Sheffield, Sheffield, UK

**Keywords:** community-based participatory research, cultural studies, disability

## Abstract

A symbiotic relationship exists between narrative imaginaries of and real-life advancements in technology. Such cultural imaginings have a powerful influence on our understanding of the potential that technology has to affect our lives; as a result, narrative-based approaches to *participatory design* (PD) of technology are an active area of investigation.

In this ongoing study, the following research questions are addressed: how can PD be optimised for the fields of robotics and assistive technology, particularly with regard to fostering empowerment and eliciting how people imagine the role of technology in their own futures? How can the symbiotic relationship between (popular) cultural imaginaries and real-life technological advancements be acknowledged within the PD process?

The study synthesises fictional inquiry and science fiction prototyping methodologies and processes over multiple workshops. Its aim is to explore and develop conceptions of robotics and assistive technology of children with *osteogenesis imperfecta* (OI, commonly known as brittle bone disease) and their families, as these populations are under-represented in collaborative research and stand to benefit from future robotics development. Narrative-based approaches are complemented by participants’ direct interaction with contemporary robots during each workshop and a ‘robot home visit’ to unite experiential understandings of robots and their current capabilities with possible futures, as well as foster mutual learning between stakeholders and designers. The study deploys a mixed methods research design with a critical posthumanist theoretical lens.

This inclusive co-designed methodology should establish a rich, nuanced picture of how people currently imagine robots in their future and facilitate all involved to deepen these conceptions. It is anticipated that everyone taking part will empower themselves to imagine fully the range of possibilities in their own personal futures in our increasingly technologised world.

## Introduction

 The inter-related fields of robotics and assistive technology (AT) have only recently begun to embrace collaborative methodologies in their research and development activities, with varying degrees of fidelity to the emancipatory spirit embodied by these. One such framework that is beginning to gain traction is participatory design (PD): the involvement of end users throughout the design process (see next section for a brief history and analysis thereof). Some studies do fulfil the Scandinavian tradition of PD’s core principles of affording all stakeholders a voice in the design of the products they use and of establishing mutual learning between designers and users (for example, Azenkot 2016 *et al*; Hamidi *et al* 2018; Lee *et al* 2017; Newbutt *et al* 2022; Rose and Björling 2017; Stegner 2023 *et al*; Šabanović *et al* 2015).

However, many studies that invoke PD or similar concepts such as co-design fail to facilitate *authentic* participation—active, sustained engagement of stakeholders instead of one or two tokenistic validation or user requirements elicitation sessions—during the robot design process. Few see it through to fruition beyond early prototypes or guideline generation for future endeavours (Stimson 2024 *et al*, in preparation). One potential explanation for these limitations is the technologically determinist and historically rarefied nature of the robotics and AT fields (Šabanović 2010). Another is the enduring disconnects between the knowledge domains and communication styles of people of different communities and roles within the PD of robots (Winkle 2021 *et al*).

The use of narrative-based techniques to bridge these epistemological and discursive gaps is a promising avenue of inquiry (Blythe 2017; Cheon and Su 2018; Dindler and Iversen 2007; Johnson 2011; Nägele 2018 *et al*; Oliver 2019; Wheeler *et al* 2018). Synthesising such arts and humanities-based techniques with those already established within science, technology, engineering and mathematics (STEM) fields, like technology immersion (Druin 1999), enables robot designers to not only create ‘better’ products but in a manner that avoids extractivism or patronising by attempting to ‘educate’ end users. It does this by facilitating the co-creation of technology *imaginaries* through storytelling, thereby taking a step towards a more equitable relationship between society and technology.

This primarily qualitative study is engaging with the work of thinkers such as Charles Taylor, Sheila Jasanoff, Despina Kakoudaki and Jennifer Rhee on imaginaries as collectively held and institutionally reinforced visions of how things should or might be in society, especially regarding scientific knowledge, social order and the relationship between the two (for a history of the concept’s use and theorisation in the Science and Technology Studies (STS) field, see McNeil *et al* (2016)).

According to Taylor (2002, 91), the (social) imaginary is ‘not a set of ideas; rather it is what enables, through making sense of, the practices of a society’. Furthermore, Jasanoff advocates for scientific and technological endeavours being conducted in full cognisance of their *co-produced* nature:

Briefly stated, co-production is the proposition that the ways in which we know and represent the world (both nature and society) are inseparable from the ways in which we choose to live in it… Scientific knowledge, in particular, is not a transcendent mirror of reality. It both embeds and is embedded in social practices, identities, norms, conventions, discourses, instruments, and institutions— in short, in all the building blocks of what we term the social. *The same can be said even more forcefully of technology*. (Jasanoff 2004, 2–3, my own italics).

This rhizomatic quality acknowledges the inextricable, symbiotic relationship between narrative imaginaries of and real-life advancements in technology (Jasanoff 2015; Wilson 2015). This notion is taken further by Kakoudaki. She explores how narrative representations of technology—specifically robots, androids and cyborgs—function as allegories that express a multitude of social and technological anxieties that have been prevalent at culture-wide levels for centuries. To name but a few, 'we have the fear of becoming automatic or mechanical, we have the fear of being enslaved, the fear of being a tool, the fear of being an object, the fear being inanimate, the fear of being rejected, the fear of being abandoned or isolated' (Kakoudaki 2014, 145). One could frame such dystopian states of existence as anti-imaginaries; how things should *not* be. These dystopian visions are potentially as useful to roboticists as a list of user requirements because what should be avoided in technology development is arguably more consequential or perilous than what should be striven for. Similarly, Rhee highlights that both fictional and real-world applications of robots reinforce inequalities across lines of race, gender and class entrenched within capitalist systems, especially in the context of labour. She duly acknowledges the ‘vast porosity’ (Rhee 2018, 6) of the boundaries of ‘technology’ and ‘culture’ and lauds the posthumanist contributions of STS scholars such as N. Katherine Hayles and Donna Haraway and those of queer theory scholars such as José Esteban Muñoz. She insists on maintaining use of the category of ‘human’ to ask deeply critical posthumanist questions:

Who is the human who is de facto valorized and normativized through the anthropomorphic visions that organize robotics? Who… is excluded, erased, dehumanized, rendered not-human? (Rhee 2018, 4).

Asking such questions in the context of PD is crucial as it has significant implications for its arguably most important aspect: equity of agency. The active involvement of marginalised groups and/or those that have not historically participated in collaborative research, and especially those who will be directly affected by technology development goes towards destabilising and undermining exclusionary and normative visions of the human and the anthropocentrism implicit in humanism. This in turn expands the scope of who—and what—has a voice in processes that will undoubtedly have transformative effects on the trajectories of, and collective well-being within, technologised futures. In other words, the ‘material consequences’ of imaginaries (Suchman cited in Rhee 2018, 4).

The concept of imaginaries has recently been used as a lens to interrogate how perceptions and assumptions influence technology design. For example, Breuer *et al* (2023) explored how robot engineers’ visions of geriatric healthcare settings affected design decisions and user engagement. During their case study, the researchers found that the participating engineers’ technocentric approach coupled with their often-simplistic conceptions of their end users’ contexts undermined the real-world applicability of their outputs. In conceiving of healthcare workers’ complex and multifarious roles as a series of tasks unaffected by local context, the engineers failed to incorporate nuanced elements of actual healthcare, such as emotional labour, collaboration and dynamic decision-making in their designs.

There was also a problematic tension seen in engineers’ valuing the domain expertise and experiential knowledge of healthcare workers, and simultaneously treating them as fearful and ignorant of technology. As the researchers explicate:

…much of the engineers’ engagement with healthcare practitioners has a strong focus toward ‘acceptance’ which is prevalent in social robotics. This notion is premised on the assumption of resistance—that healthcare workers hold irrational fears of robots based on insufficient understanding of the technology; it suggests that an important goal in engaging with healthcare workers is to convince them of robotic solutions (Breuer *et al* 2023, 23).

This narrow conception of user engagement ‘implies a one-way flow of knowledge from the engineering team to the participants, with limited room for the participants to express their perspective’ (Breuer *et al* 2023, 25). While engineers do often invite domain experts to share their insights within the human-computer interaction (HCI) field, these endeavours are constrained in their utility by ‘the dominance of acceptance approaches’ (Breuer *et al* 2023, 25).

These two issues in robotic and AT development—the weak grasp of end user contexts and the narrow conception of user engagement as acceptance gained through unidirectional education of end users—can be addressed using PD. The point of departure of this ongoing study is to use sociotechnical imaginaries grounded in popular culture as an output of PD intervention. Encapsulated in short science fiction stories that are currently being collaboratively crafted by robotics researchers, workshop facilitators and potential end users of robots and AT, these narratives will act as a *lingua franca* between disparate stakeholder groups possessing sometimes vastly different knowledge domains and lived experiences. This is predicated on the idea that popular culture influences sociotechnical imaginaries (e.g. in the West, scientists and laypersons alike are at least aware of conceptions of robots informed by the range of robots featured in the media franchise *Star Wars*). Using already shared frames of (popular) cultural reference will enable all stakeholder groups, regardless of knowledge domains, communication styles or technical literacy to establish mutual understanding and consensus in technology design processes. Applying the concept of imaginaries in this way should establish a shared, accessible vision for what stakeholders collectively do and do not want from these technologies. The resulting co-designed methodology will thereby facilitate future technology development that is both equitable and applicable in real-world contexts.

Narrative imaginings such as science fiction have a powerful influence on our understanding of the potential that technology has to affect our lives. As stated, narrative-based approaches to PD of technology are an active area of investigation. The most prominent example to date is Brian Johnson’s science fiction prototyping, a pragmatically oriented methodology for using science fiction to explore ‘implications, effects and ramifications of [that] science or technology’ on multiple levels (Johnson 2011, 2).

Another salient example is fictional inquiry, a Scandinavian tradition-inspired technique that ‘creates partially fictional settings, artefacts and circumstances through a shared narrative’ (Dindler and Iversen 2007, 214). This technique allows the fiction to move away from how things currently are in real life, but has participants play as themselves; crucially, not assuming a fictional role/character persona. Having people play as themselves in a fictional scenario facilitates the bypassing of ‘existing sociocultural structures’ (Dindler and Iversen 2007, 213) (such as parent/child or carer/patient dynamics), thereby enabling participants to express themselves more freely, which yields a greater understanding of their conceptions and attitudes.

The outcomes of this collaborative, analytic and culturally sensitive undertaking will be co-crafted narrative visions of the future that can be used to both inform technological advancements and empower people to understand how these might affect them, their loved ones and the world around them. The relative absence of robots and AT in everyday life remains a hindrance to the general public’s understanding of what such technologies can and cannot do, and what they might be capable of in the future. The marrying of technical knowledge with imaginative potential afforded by storytelling-based PD is crucial to producing better outcomes for all stakeholders, whatever form these might take.

This paper is unorthodox for this publication in that it primarily focuses on methods and methodology, as opposed to presenting the full results of a completed study. Furthermore, the study itself has a meta-quality; rather than asking what *x* population thinks about topic *y*, it seeks to proffer a practical, context-sensitive framework for designers to use *with* their own stakeholders in order to enact equitable robot development. Its contributions deliberately include unvarnished reflections on the process of designing and conducting the study thus far, and it harmonises concepts and practices from axiologically diverging fields.

Due to the necessarily rigorous nature of minimising the risk of patients coming to physical, mental or emotional harm through involvement in research, the process of obtaining full National Health Service (NHS) ethics approvals to work with the target population took a full year. At the time of writing, the study is not yet complete, with the robot home visits and a final workshop still to be undertaken.

As such, the purpose of this contribution is threefold:

1. To provide an overview of PD and its theoretical foregrounding for the study design and its intended outcomes.
2. To share candid reflections on the NHS ethics approval process, contributing insights on how it could be improved to facilitate participatory research.
3. To present interim findings thus far.

## Theoretical background

The study is informed by several areas of emancipatory theory and praxis, including critical disability studies, feminist human-robot interaction (HRI) and emic ethnography. For brevity purposes—and so as not to repeat concepts or histories that are more salient to other contributions in this Special Issue—overviews of the areas that pertain strictly to the PD methodology being co-designed are given in this section.

### Scandinavian participatory design

Given its roots in the various social, political and civil rights movements in the 1960s and 1970s, PD has always been both inherently and expressly political in its aims (Simonsen and Robertson 2012). In particular, the Scandinavian tradition of PD champions ‘an unshakable commitment to ensuring that those who will use information technologies play a critical role in their design’ (Simonsen and Robertson 2012, 2).

PD is not defined by a specific set of rules or methods, but by a commitment to its two core principles:

- Enabling all who would be affected by a product/service to have their voice heard, regardless of their ability to ‘speak the language of professional technology design’ (Simonsen and Robertson 2012, 2).
- Facilitating 'a process of mutual learning for both designers and users can inform all participants’ capacities to envisage future technologies and the practices in which they can be embedded', and serve to enable laypeople to be able to define what they want from a design process (Robertson and Simonsen 2012, 3).

This pursuit of equity and empowerment is seen in the early projects that came to define Scandinavian PD in the 1970s. Collaborations between researchers and trade unionists, these endeavours treated ‘democratic participation and skill enhancement’ (Ehn 2017, 41) as valid and desirable ends in and of themselves, rather than using PD merely as a means to designing a better product or increasing worker productivity. In the face of management-driven technological change, the practitioners made a conscious and hitherto unprecedented decision to uphold the interests of workers—those who would be directly affected by the new computerised systems being imposed—over those of company bosses. It was pernicious attempts at task automation and de-skilling employees across industries as a method of worker subjugation, in combination with wider societal changes and a political milieu unique to Scandinavia that were instrumental to the evolution of PD as it is understood today.

As stated, the commitment to side with marginalised communities is attributed in part to societal changes occurring at the time. Increases in citizen engagement at local levels in Western European countries, along with geopolitically seismic events such the Vietnam War, led to a paradigmatic transition in IT design (Simonsen and Robertson 2012). Traditional design textbooks had long advocated a waterfall model where ‘problems were defined by management with no input from those who would be using the system’ (Simonsen and Robertson 2012, 23). Over time, designers began to try to ‘capture’ the cognitive process of individual users (e.g. by tracking keystrokes and eye movements) and transpose it into program interface and workflow designs (Simonsen and Robertson 2012). This well-intentioned line of inquiry can be considered a liminal stage in the journey from top-down towards (the attempt to establish) equal power distribution in IT design, as it was part of the emergence of the concept of ‘users’, which had had no place in a system where large mainframe systems were custom-designed for individual companies at the behest of management. Such efforts eventually gave way to an understanding that technology is deeply bound by the social and political contexts in which it is used, as opposed to formalised best practices. This understanding is at the heart of what is known as sociotechnical design (a history of which can be found in Mumford (2006)), a pioneering philosophy that comprised a set of methods revolving around the central tenet that the improvement of working conditions and the advancement of human knowledge are equally as important as system design (note that this ties in well with critical theory). It was originally developed by the London Tavistock Institute and spread far beyond England, and notably to Scandinavian employers’ associations (Simonsen and Robertson 2012). The approach was heavily critiqued by Scandinavian researchers at the time for its narrow approach to worker participation, ‘as informants in a process dominated by managers and their specialists’, and for its naïve conception of power distribution, as it advocated a voting system for decision-making in an environment with an unequal balance of authority (Simonsen and Robertson 2012, 25). However, it was undoubtedly a strong influence on PD, lauded for the fact it emphasised the importance of attending to organisational concerns as well as technical ones, and for introducing prototyping onto the design scene (Simonsen and Robertson 2012).

This transition speaks to the epistemological and motivational tensions between designerly expertise and, if you will, ‘userly’ expertise, and the productive shift from a user-centred design approach (where design is done *for* users) to a PD approach (where design is done *with* users), particularly within Scandinavia. The elicitation, respecting and synthesis of these historically disparate knowledge domains constitutes both the art and the ideology of PD, which, as stated, has its roots in the unique political milieu of Scandinavia. Namely, an unusually strong tradition of trade unionism, which led to explicit legislation affording workers the right to ‘information and some degree of co-determination over the conditions of their work’ (Simonsen and Robertson 2012, 25).

Workers in many industries were naturally concerned by the impending threat of de-skilling and computerisation, and it was worker criticism of a lack of practical relevance in the original conception of the NJMF project that is widely regarded as the impetus for a progression from a user-centred to a PD approach in Scandinavia and beyond (Simonsen and Robertson 2012). Although part of efforts to uphold workers’ interests and agency in the leadership-enforced computerisation process, the NJMF project was originally planned to be much more traditional in approach; researchers would conduct analysis into the workplace environment without active worker participation. The trade unionists made the apt observation that, while the project was interesting, ‘it was not possible for the shop stewards and members to find any connection between our work and what they could do in direct, everyday practice within the local unions and the Metal Workers Union’ (Nygaard and Bergo 1974, 6). On hearing this criticism, the researchers 'realized that we were likely to write a series of reports which would remain unused, perhaps also unread, on the bookshelves of the shop stewards' (Nygaard and Bergo 1974, 6).

This revelation was no doubt disheartening but ended up being crucial to the establishment of PD, as it resulted in the application of action research practices in the project. The project’s emphasis was now on worker agency and action; the end goal being the building of worker knowledge, voice and technical vocabulary to empower the metal workers to hold their own in negotiations with management on the introduction of computer systems into their workplace. It was decided that the project’s results would chiefly take the form, not of papers and reports, but of worker *action* at either local or central levels (Nygaard and Bergo 1974).

The early mistakes and resulting changes within the NJMF project are regarded as the genesis of a key argument for PD, which has a political aspect (‘…people *should have the right* to influence their working conditions’ (Simonsen and Robertson 2012, 27, their italics) and a pragmatic aspect (‘…learning between and among the different power groups’ (Simonsen and Robertson 2012, 27)). The lead investigator of the project, Kristen Nygaard, soon began to inspire other Scandinavian researchers in the field, including influential PD practitioners Pelle Ehn (responsible for the seminal UTOPIA and DEMOS projects) and Morten Kyng (responsible for the seminal DUE project) (Sundblad 2010).

### Participatory design with marginalised communities

The literature shows that PD principles and commonly employed methods, such as the use of prototypes, mock-ups and generative tools (Sanders 2000), and frameworks such as ethnographic (Crabtree (1998); Crabtree *et al* (2000); Moline (2021)) and narrative-driven (Dindler and Iversen (2007); Nägele (2018) *et al*) have been successfully used to explore potential futures across many contexts and with different user groups. These range from new technologies in the workplace (as in the aforementioned seminal PD work) to education contexts (Cumbo and Selwyn (2022); Druin (1999); Cumbo (2019)), municipal and species-wide issues (Moline (2021)) and with adults and children in collaboration (Yip *et al* (2017)), and people of various age groups with special needs, disabilities and/or neurodivergence (Malinverni *et al* (2014); Seale *et al* (2021)).

Given the strong emphasis on empowerment in PD, there naturally exists a wealth of prior work on PD with marginalised communities. These range from the development of frameworks for and case studies working with people of colour, women, lesbian, gay, bisexual, transgender, queer, and questioning (LGBT+) individuals, economically disadvantaged urban and rural communities, socially disadvantaged communities, (historically) colonised communities, people with special needs, disabled people, children and older adults. There is much crossover and variation with regard to conducting PD with people having separate and intersecting marginalised attributes and the outcomes pursued, from designing leg prostheses with physically disabled children in rural Cambodia (Hussain (2010)) and designing do-it-yourself (DIY) AT with diverse stakeholders in Western Kenya (Hamidi *et al* (2018)), to using PD to understand how LGBT+ people living in rural parts of the USA experience community and technology (Hardy and Vargas (2019)) and creating a distributed participatory design platform to design a video game with deaf children (Galvão *et al* 2021).

Consequently, there is much work on critical and decentralising perspectives in PD, such as decolonisation (Ray Murray *et al* (2021); Seppälä *et al* (2016)) and de-anthropocentralisation (Akama *et al* (2020); Forlano (2017); Hall *et al* (2006); Rice (2018); Wakkary (2020)). Furthermore, questions of inclusion, ethics and how to manage the balance between enabling participation and protecting vulnerable individuals from harm within the PD process continue to be active areas of investigation and debate (Antle and Hourcade (2022); Iivari *et al* (2022); Read *et al* (2013); Read *et al* (2014); Spiel *et al* (2020)). These aims and concerns are in keeping with the notion of entanglement currently being used in the fourth wave of HCI (Frauenberger (2020); Porayska-Pomsta *et al* (2012)), which embodies an avowedly holistic, posthumanist perspective.

While neurodivergence is not the same as disability, the two certainly can and do overlap, and their demarcations often blur. The specific qualities and needs of different manifestations of neurodivergence and disability have evidently been treated as requiring highly specified sets of PD methods and resources. However, the PD field is replete with PD frameworks, methods and toolkits targeted at a range of different communities and technology-related outcomes.

As such, rather than creating an entirely new methodology, valuable theoretical and practical contributions can be made by synthesising and modifying existing techniques and guidelines. The aim of this is to improve their accessibility by a range of marginalised communities through addressing already identified and emerging limitations. Relevant examples include: Benton *et al* (2012), Bayor *et al* (2021), Guha *et al* (2013), Drain *et al* (2021), Porayska-Pomsta *et al* (2012), Read *et al* (2013), Iversen *et al* (2018), Dindler and Iversen (2007), Bertel *et al* (2013), Jussli and Gewald (2021), Shahin *et al* (2021), Crabtree *et al* (2000), Nägele *et al* (2018).

## Methodology

### Patient and public involvement

The patients (children with OI and their immediate families) were involved from the beginning of the study (January 2024). In accordance with striving for equitable involvement of (potential) end users in the design of products and services they will use, it would have been ideal to co-design the entire study with the families from the outset. However, given the institutionally instigated nature of predefined PhD projects, the associated milestone timelines and the need for the NHS to have a complete understanding of proposed research before approvals can be granted, this was not possible.

Instead, the study was designed to be inherently flexible, focusing on transferring agency as and when desired by participants and on practising *micro-ethics* within sessions (Spiel *et al* 2020). For example, giving participants a choice of different modes of engagement with PD activities, the choice not to participate in a particular activity and to determine the length and depth of engagement with each activity. The research questions and outcome measures were not developed in accordance with the explicitly expressed priorities, experience nor the preferences of the patients. They were, out of necessity, informed by the researcher’s (my) perspective on participants’ priorities, experience and preferences. Using insights shared by a Senior Clinical Psychologist at Sheffield Children’s Hospital, the study was designed to involve a population that is under-represented in collaborative research. In attempting to adopt an *emic* (insider’s) perspective, and to advocate for and facilitate the agency of patients, the research questions are deliberately oriented not towards extracting a list of user requirements—as would be expected when adopting the more technocentric, *etic* (outsider’s) perspective of a designer or programmer—but towards maximising the value of patient involvement in the PD process.

Given the age and vulnerability of the child patients, as well as to avoid burdening the families, they will neither be involved in analysis beyond self-reflection of the study’s thematic content, nor be involved in the dissemination of the study’s results. This is to protect the children’s rights and assent, as well as to avoid them feeling as if they are being assigned homework or perhaps even being asked to do my doctoral work for them. The age/developmental status of the children is another factor, as RTA is necessarily long, repetitive and therefore likely tedious to children. The parent/carer participants will be supplied with the full published results once the study is completed to read and explain to the child participants, and I will be available to answer any questions and for any follow-up discussion, if the participants so desire.

### Study design

This study is using mixed methods to explore current understandings of robots and possible futures involving them. This reflects the interdisciplinary nature of the imagining technologies for disability futures (itDf) project, uniting STEM and arts/humanities techniques to take a step towards bringing the historically rarefied robotics field closer to the needs of end users. This choice was made to incorporate the disciplinary tradition of robotics as well as that of (Scandinavian) PD and my own academic experience to date. While the study’s research aims are primarily abstract and emancipatory (facilitating empowerment and deep engagement with potential futures, as opposed to extracting design ideas or user requirements), the resulting PD methodology must have real-world relevance with regard to actualising the creation, testing, iteration and manufacturing of physical prototypes, products and services.

At a lower level, this study aims to contribute novel knowledge to the robotics field by collaboratively crafting with disabled children a PD methodology tailored for use in future development of robotics and wider AT. It will therefore address the following research questions:

- *RQ1*: How can PD be optimised for the fields of robotics and AT, particularly with regard to fostering empowerment and eliciting how people imagine the role of technology in their own futures?
- *RQ2*: How can the symbiotic relationship between (popular) cultural imaginaries and real-life STEM advancements be acknowledged within the PD process?

The qualitative methods comprise reflexive Thematic Analysis (RTA) (Braun and Clarke 2021b) of workshop audiovisual recordings, resulting co-produced fictional artefacts and participants’ reflections on the robot home visit. These will be complemented by quantitative sliding scales regarding attitudes towards robots before the PD process commences, attitudes towards the robots featured throughout and attitudes regarding the efficacy of the PD methodology itself.

At the end of the study, the generated themes will be compared with the sliding scale responses to evaluate the co-produced PD methodology’s efficacy at developing participants’ consideration of technology futures, and, therefore, how optimised the methodology is for the fields of robotics and AT, answering *RQ1*.

The thematic analyses and sliding scale responses will then be compared using their mean average values. This will establish a holistic depiction of participants’ changes in and depth of imaginaries of their future involving technology, answering *RQ2*.

#### Data collection and analysis

This section details further the methodological choices made in this study.

The data types being collected are: audiovisual recordings of all preparatory video calls and workshops, the co-crafted short stories, semi-structured interviews and self-reported diary entries from the robot home visit, sliding scale responses throughout the process on attitudes towards the robots featured and the process itself.

The study is employing mixed methods, using a concurrent transformative design type (Hanson *et al* 2005). This type prioritises qualitative data over quantitative, both with regard to primacy and amount to be collected and analysed during the project and its underlying ontology, epistemology and axiology: a critical-ideological paradigm, specifically contextualism.

The method of data analysis is Thematic Analysis (TA). TA is a way of identifying patterns in qualitative data in order to answer questions about people’s views, perceptions and representations of a phenomenon. As the itDf project is concerned with how people imagine (their views and perceptions) and cultural imaginaries (representations) about possible futures (a phenomenon), TA is a natural choice.

The reflexive approach to TA (RTA) espoused by Braun and Clarke (2021b) is particularly appropriate owing its methodological and philosophical kinship with both PD and ethnography. It is by definition iterative and recursive, and constantly mindful of researcher position and subjectivity (their position within the academy, intellectual, philosophical and methodological dispositions, implicit and explicit biases, etc.) and their inevitable effects on participants, data and the conclusions drawn from it (Braun and Clarke 2021b). It demands that theoretical and epistemological assumptions are made explicit and continually reflected on by the researcher(s) for their moulding and curbing effects. It rejects outmoded pretences of the possibility of truly ‘objective’ data or research, such as those forwarded by logical positivism and scientism; it is avowedly interpretivist in its stance. It is also what is known as ‘Big Q’ as opposed to ‘small q’; ‘fully qualitative’, or qualitative in both techniques and underlying values. That is to say, it includes ‘a conceptualisation of researcher subjectivity as a resource for research and of meaning and knowledge as partial, situated and contextual’ (Braun and Clarke 2021a, 39), as opposed to one where qualitative methods are used in the service of values that hold ‘objective, generalisable, reliable and replicable knowledge as ideal’ (Braun and Clarke 2021a, 39).

Furthermore, its proponents note that ‘many reflexive TA researchers do indeed have some kind of social justice motivation, be it “giving voice” to a socially marginalised group, or a group rarely allowed to speak or be heard in a particular context, or a more radical agenda of social critique or change’ (Miller and Brewer 2015, 849). It is understood as being located within the ‘phenomenological or experiential qualitative research tradition’, which is ‘centred on the exploration of participants’ subjective experiences and sense-making’ (Braun and Clarke 2021a, 39). Given this project’s roots in critical theory, the Scandinavian PD tradition, and the disability/marginalisation of its participants and some of its co-investigators and mentors, this further boosts RTA’s suitability for achieving the itDf project’s emancipatory aims.

### Co-design process

This section details the ongoing co-design process and provides reflections on the steps taken thus far.

#### University and NHS ethics approvals procedures

The University of Sheffield’s research ethics approval procedure went smoothly and swiftly, with actionable feedback given and a final decision made within 10 days. Its policies, requirements and the Ethics Application System website’s user interface were clear and easy to navigate.

Conversely, the NHS ethics approval procedure took far longer than originally anticipated, even considering its reputation and cautions from colleagues and friends regarding its length and complexity. This was due in no small part to how unfriendly, and frankly, how antiquated the Integrated Research Application System (IRAS) website’s user interface is. Setting aside the website’s clunky design, although each question in the application form webpages included a tooltip with additional information on how to answer it, these were overly brief, vague and used technical jargon that would be virtually inscrutable to anyone not well-versed in NHS processes. None of my three PhD supervisors had any prior experience of the IRAS, nor of working with the NHS. As such, attempting to identify, contact and receive a definitive answer from appropriate University or NHS staff members on resolving various roadblocks encountered throughout, as well as sourcing and completing the many supporting documents required prolonged an already convoluted procedure. Even basic questions such as whether the study required a full or only a partial HRA review could not be definitively answered by automated questionnaires in place that are supposedly designed to streamline the IRAS application process.

From start to finish, the full NHS ethics application process took 12 months; the entirety of year 2 of the PhD. It is a fact that the choice to involve NHS patients has significantly elongated the PhD timeline and, therefore, the ability to present a completed study in this Special Issue. I acknowledge the necessity of the NHS ethics approval procedure being meticulous to ensure that patients come to as little physical, emotional and mental harm as possible through any involvement in research. However, from direct experience of working with NHS systems, I understand why (from anecdotal evidence) many researchers, even those much further along in their academic careers, avoid working with NHS populations due to the length and complexity of the ethics procedures and tools involved.

This particular study was always non-clinical in nature and relatively low-risk from an ethics perspective. At its core, it is about families having fun with robots and writing short stories about them. The only noteworthy risk comes from the localised context; of people with brittle bones tripping over small, commercially approved robots that exhibit limited or no spontaneous movement. There is also the potential for discussing emotive or sensitive topics with other people with similar lived experiences to cause distress; however, this situation is more likely to encourage open, convivial conversation.

As such, the time and effort it took to obtain NHS ethics approval for this study was excessive. Protracted timelines and burdensome requirements could certainly discourage valuable research from being undertaken with NHS patients, who might have much to both offer and gain from involvement in lower-risk research—especially in participatory or other non-traditional research.

Nevertheless, the ideologically motivated decision to involve a disabled demographic—especially one that is under-represented in participatory research—was both appropriate for this study and consonant with the broader aims of the itDf project. The study’s eventual contributions will almost certainly be all the more valuable for it, from both academic and disability activist perspectives. Setting aside concerns surrounding meeting institutionally defined goals to earn a PhD to a particular schedule, simply witnessing families enjoy themselves while they engage with possible technology futures as a result of a study that aims to neither hyperfocus on nor ignore their lived experiences of disability, is proving immensely rewarding.

I hope that these small insights into the NHS ethics approval procedure from a non-clinical PhD researcher’s perspective might encourage some change within it to make it less onerous for researchers at all levels to work with NHS patients, especially in lower-risk studies. As identified earlier, aspects that could be improved include modernising the user experience of tools such as the IRAS website, and providing clearer and more layperson-friendly information on how to successfully navigate the NHS ethics approval process.

#### Participant recruitment

A Senior Clinical Psychologist at Sheffield Children’s Hospital, Dr Rebecca Jones, agreed to act as facilitator between myself and the target population. Dr Gemma Wheeler, a Senior Project Manager with a background in Design Research, based at the National Institute for Health and Care Research HealthTech Research Centre in Paediatrics and Child Health, also offered assistance regarding the design and practical implementation of the study as she has extensive experience of conducting co-design workshops with children with chronic health conditions. Jennifer Lomas, a Research Assistant at Sheffield Children’s NHS Foundation Trust, also assisted with recruitment by explaining to potential participants the project and acting as a point of contact for the families. I extend my thanks to all involved for their expertise and support in this process.

I had originally intended to select a range of participants to reflect the diversity within the target population from the pool of recruitment call respondents, given that OI affects both sexes and all ethnicities equally. This was not possible due to the relatively small size of the demographic and correspondingly low number of families who answered the recruitment call. It is serendipitous, then, that the resulting cohort (at least, the child with OI participants) was diverse with regard to ethnic background, and equal with regard to sex representation: two males and two females, with each family having a different ethnic or cultural background (exact groups are not stated here to protect participants’ identities).

The participant recruitment process took longer than anticipated, likely due to the restrictive nature of the original age range specified inclusion and exclusion criteria. For both the children with OI and any similarly-aged, non-disabled siblings, these criteria were as follows.

- Inclusion criteria:
  - Aged between 12 and 14 years.
  - Willing and able to commit to a 6-month PD project including both in-person and virtual involvement.
  - Homogeneous with each other in terms of cognitive ability to encourage group bonding.
  - Any gender identity, ethnic background or other sociodemographic characteristics.
  - Able to speak fluent English.
  - Able and willing to commit to travelling to The University of Sheffield once a month, health and other circumstances permitting.
- Exclusion criteria:
  - Outside of the specified age range.
  - Unable or unwilling to commit to a 6-month PD project including both in-person and virtual involvement.
  - Unable to speak fluent English.
  - Unable to commit to travelling to The University of Sheffield once a month, health and other circumstances permitting.

The age range of the child participants was expanded to 11 and 15 years, which yielded a quorate number of families.

Participants were reimbursed for reasonable travel expenses to and from the university campus and provided with a complimentary lunch before each in-person workshop.

#### Video call 1: icebreaker

As a precursor to in-person sessions, an online video call between the families, myself, and a facilitator (thanks again to Dr Wheeler) took place, using The University of Sheffield’s preferred platform, Google Meet, which was recorded with consent. This minimised travel burdens and allowed everyone to get to know each other in a COVID-19-safe manner. The purpose of the video call was to establish rapport and to provide information on the purpose of the study and its wider project context. Specifically, Dr Wheeler and I explained exactly why participants are engaged in the co-design project and what changes it might inspire, thus establishing a clear understanding that this study will not seek to develop a product or prototype. In common sense and child-friendly terms, 'we are not designing or building a robot, but together we are going to come up with ways of giving ideas to people in the future who will make robots for people with your condition'.

The video call also fostered realistic expectations regarding levels of participation/engagement and initiate a study-long attempt to ensure that participants and their contributions to be meaningful rather than being used in a tokenistic, or worse, in an ableist or pathologising manner.

In addition to setting expectations, this video call was designed to encourage participants’ excitement about the workshops, and collect initial qualitative data regarding existing understandings and desires surrounding robotics, by asking questions (such as “what do you imagine the robots we’re going to bring you are like?” and “what would you like them to do?”). The verbatim utterances during all video calls were thematically analysed in accordance with Braun and Clarke (2021b) (see section ‘Interim findings’).

#### Video call 2: robot drawings

At the end of the first video call, participants were asked to submit drawings of robots created by them using whichever form they prefer (whatever digital software or traditional implements they have at their disposal). The use of drawing is considered appropriate for the age range of the children involved (G. Wheeler, personal communication, 2021). In accordance with intergenerational co-design expert Allison Druin’s tailoring of activities according to each demographic’s preferences, both child and adult participants were given the option of producing a detailed description in lieu of a drawing (Druin 1999).

Three out of four families participated in this session as one family could not attend due to unforeseen circumstances. At this stage, only three children with OI were involved, meaning no siblings without OI were involved. Only two of the children produced a creative artefact in advance of the session: one child created a detailed drawing of a robot they would like to see in the future using a tablet, and the other chose to write a detailed description thereof.

#### Workshop 1: help MiRo get home!

The first workshop was held in The Diamond, an accessible and suitably futuristic-looking venue at The University of Sheffield. In the session, participants had free, undirected contact with Consequential Robotics' companion animal robot MiRo (in ‘demo’ mode, with the ‘sleeping’ behaviour turned off to avoid boredom in participants) and then were presented with the basic narrative premise of helping a lost MiRo and its way home to its ‘herd’. The narrative was framed using a physical maze erected ahead of time in the venue, with the MiRo being lost within it. Rather than furthering a sense of animism in the robot (or obscuring a human operator using the ‘Wizard of Oz’ technique often used in HRI research), participants were overtly invited to take turns controlling MiRo using web interface-based teleoperation and/or a Sony PlayStation DUALSHOCK 4 wireless controller, guiding it through the maze until it joined the rest of the herd. This aimed to foster an experiential understanding of what this particular robot can and cannot do and provide an opportunity for the faciiltators to ask questions regarding personhood in relation to technology ('how does seeing through MiRo’s eyes make you feel?').

Participants were then be invited to break out into small groups and flesh out the characteristics, habits and ‘world’ of MiRos as a ‘species’. They were provided with writing implements and art supplies to facilitate this process. The overall objective of this session was maintaining an emphasis on explicating the robots themselves without any relationship to humans, caring or otherwise. It was expected that this would allow the children with OI to immerse themselves in the fiction, without trying to bring attention towards their symptoms or ‘problems’. This was to set the tone of the sessions, one of fun and creativity: crucially, not something tokenising or pathologising.

Everyone then reconvened to discuss their ideas and integrate them into a simple story, with support from a professional illustrator (thanks to Chris Redford at Nifty Fox Creative) to create an ‘island mural’ of their ideas and narrative timeline using the fictional inquiry notion, as employed by Wheeler *et al* (2018). The rationale for this was that it would help participants to feel that everyone is having their ideas listened to, and to document narrative progression that can be elaborated on in future sessions.

The session was very successful overall, despite a technical issue with the MiRo robots at the beginning, which was swiftly resolved (thanks to Aung Htet, Research Assistant at the University of Sheffield and PhD Candidate at Sheffield Hallam University). The families seemed to enjoy themselves, and the quality and depth of discourse surrounding future technologies was similarly inspiring as it was during the video calls. The verbatim utterances and co-created fictional artefact are in the process of being thematically analysed and will be presented in a future article.

#### Workshop 2: the MiRo homeworld

The purpose of the second workshop was to build on the fictional world created in the first, and then bring participants into the narrative as themselves. This complies with the fictional inquiry process (Dindler and Iversen 2007) and allows for a natural, unforced connection between robotics, science fiction and the lived experiences of participants. With help from a VR telepresence experience (thanks again to Aung Htet and to Daniel Camilleri, Founder and Chief Technology Officer at BOW) to aid immersion, participants were asked to breakout into small groups and imagine they are visiting the homeworld or island of the MiRos that they created in the first session. They were asked questions regarding how they might introduce themselves to the robots, and how they might be treated as visitors, then as neighbours. They were then be asked the reverse: 'What would happen if these MiRos came to our world?', 'If a MiRo came to live with you, would you like that?' The intention here was to encourage imaginaries regarding social robots in their own lives.

As in the first session, the workshop culminated in a group discussion, this time with MiRos depicted in our world and participants’ homes. At the end of the workshop, participants were asked if they would like for a MiRo robot to come and visit them for a couple of hours (parental/carer consent was sought in advance).

As with workshop 1, the verbatim utterances and co-created fictional artefact are in the process of being thematically analysed and will be presented in a future article.

#### Robot home visit

The robot home visits are currently being scheduled with the families. The robots will act as probes; objects which are sent ‘into the everyday life of people to collect information’ (Jarke and Gerhard 2018, 137) with little instruction or direction. This enables participants to deepen their experiential understanding of a robot in a more naturalistic manner. It also allows for unobtrusive research to be undertaken. After a period of free, undirected contact with the chosen robot, I will collect more verbatim data, asking questions regarding their experience with the robot and its perceived desirability and usefulness.

As an emic ethnographic study would be difficult to conduct, given that the participant group has been artificially brought together and do not have regularly shared environment in which they can be observed, the use of cultural probes in participants’ respective homes will allow for the collection of more ecologically valid, participant-led data. The results of these probes—qualitative data, in a form of the participants’ choosing—will also undergo RTA and be included in mixed methods comparison at the end of the study, as described earlier.

#### Workshop 3: my robot friend

The third and final workshop will introduce some contemporary assistive technology (a telepresence robot) and revolve around discussing participants’ experiences with the robots in their home. As well as sharing each other’s feelings of their time with the robot, more practical and future-oriented questions will be asked, such as ‘did the robot get in the way?’, 'do you think the robot could help you in day-to-day life?' and 'how could the robot change to be a better friend to you?' This is intended to be a sensitive way of introducing how robotics might feature in the lives of children with OI into discussions without overtly focusing on the condition and the ‘problems’ it causes.

This session will also act as a debriefing session. It will give participants space to reflect more deeply on their conceptions of robotics and AT and to evaluate the co-designed methodology and its efficacy as a means of establishing mutual learning between designers and users. Participants will ‘compare notes’ and continue deepening each individual’s considerations of robotics and participatory design skillsets.

## Results

This section presents the interim findings of the study thus far as an indicator of the kind of actionable outcomes designers and their stakeholders could expect from the methodology being co-designed. The data included covers video calls 1 and 2 only. Given the study’s protracted timeline and the recursive and iterative nature of RTA (Byrne 2022), the analysis presented here should be considered partial, preliminary and subject to refinement with further accumulation and analysis of data (as always, one might argue, when operating in an interpretivist-constructivist research paradigm). The data yet to be collected will inevitably shed new light on already collected data and thereby deepen the insights generated (Byrne 2022).

### Interim findings

Initial generated themes thus far comprise the following:

- ‘Robots should embody both organic and inorganic qualities’.
- ‘Robots should facilitate (not hinder) human-human communication’.
- ‘Robots should take on work or tasks that humans will tire of, are at risk of harm from or would become dangerous or inefficient at’.
- ‘Robots are considered exciting and cool by virtue of their futuristic, novel aesthetic’.
- ‘Robots should be predictable in their functionality, presentation and communication style’.
- ‘Robots should help disabled people and other people with access, participation or health issues’.
- ‘Robots can be used in educational contexts’.
- ‘Popular (Western) culture provides a valid blueprint for future robots’ functionalities and morphologies’.
- ‘Robots should offer companionship in a similar manner to the human-animal relationship’.
- ‘The category of “robots” also encompasses autonomous entities that do not mimic living beings nor their morphologies’.

The raw qualitative data, first coding iteration and provisional themes generated are included in the online supplemental appendix. They are colour-coded to indicate which data items were grouped into which provisional themes. Given the study’s timeline, a full exegesis of the data and the ‘thematic story’ of the data (Braun *et al* 2019) thereof will be published in a future journal paper.

### Discussion

The co-crafted short stories, and the themes generated from both the stories and verbatim utterances within PD sessions constitute a holistic picture of participants’ conceptions of possible technology futures. This picture can be understood across disparate stakeholder groups by virtue of their foundation in shared cultural imaginaries. It can be operationalised by STEM (technical) stakeholders' transposing the desiderata present within into user requirements that meet their end users’ needs and desires. Crucially, these requirements are collaboratively defined *with* non-STEM stakeholders through their *authentic* participation in the design process.

The data collected thus far indicates an active engagement with and openness on the part of all participants towards the prospect of incorporating robotic and assistive technologies into their lives. Analysing the affective quality of verbatim data from in situ interactions as well as sliding scale responses to the enjoyment and efficacy of the PD process over the course of the study should yield a fit-for-purpose PD methodology that will enable future technology development endeavours to better meet a variety of stakeholders’ needs and desires. This would improve user acceptance and long-term adoption rates as well as have important implications for social justice.

Other PD projects might find value in this study owing to its commitment to equity of stakeholder agency and grounding PD sessions and their outputs in both imagined and experiential understandings. The combination of storytelling with direct contact with real contemporary robots addresses non-technical stakeholders’ common misconceptions of the nature and current possibilities of robots and AT. It also encourages technical stakeholders to actively participate in collectively imagining how such technologies might and should—and should not—be in the future, and so meet their end users in the middle as opposed to trying to educate or convince them.

## Conclusion

This paper has presented an ongoing study that is co-designing and evaluating an equitable PD methodology for involving non-technical stakeholders in robot and AT design processes. Rather than developing an end product or prototype, the emphasis is on fostering the Scandinavian PD tradition’s democratic values and enduring social benefits (encouraging equitable relationships and healthy self-esteem; creating learning and development opportunities). The resulting methodology will facilitate the means to collaboratively create a rich, nuanced picture of how people currently imagine robots and other emerging technologies in their future, and to develop this conception. It will thereby establish a common design parlance between developers of their stakeholders, leading to the creation of products and services that better meet the needs and desires of their intended end users.

## supplementary material

10.1136/medhum-2024-013039 Supplementary file 1

## Data Availability

All data relevant to the study are included in the article or uploaded as supplementary information.

## References

[R1] Akama Y., Light A., Kamihira T. (2020). “Expanding Participation to Design with More-Than-Human Concerns.”.

[R2] Antle A. N., Hourcade J. P. (2022). “Research in Child–Computer Interaction: Provocations and Envisioning Future Directions.”. International Journal of Child-Computer Interaction.

[R3] Azenkot S., Feng C., Cakmak M. (2016). “Enabling building service robots to guide blind people a participatory design approach.”.

[R4] Bayor A. A., Brereton M., Sitbon L., Ploderer B., Bircanin F., Favre B., Koplick S. (2021). “Toward a Competency-Based Approach to Co-Designing Technologies with People with Intellectual Disability.”. ACM Transactions on Accessible Computing.

[R5] Benton L., Johnson H., Ashwin E., Brosnan M., Grawemeyer B. (2012). “Developing IDEAS: Supporting Children with Autism within a Participatory Design Team.”.

[R6] Bertel L. B., Rasmussen D. M., Christiansen E. (2013). “Lecture Notes in Computer Science (Including Subseries Lecture Notes in Artificial Intelligence and Lecture Notes in Bioinformatics) 8118 LNCS (PART 2).”.

[R7] Blythe M (2017). “Research Fiction: Storytelling, Plot and Design.”.

[R81] Braun V., Clarke V. (2021a). “Can I Use TA? Should I Use TA? Should I Not Use TA? Comparing Reflexive Thematic Analysis and Other Pattern-Based Qualitative Analytic Approaches”. Counselling and Psychotherapy Research.

[R8] Braun V., Clarke V. (2021b). Thematic Analysis: A Practical Guide.

[R9] Braun V., Clarke V., Hayfield N., Terry G., Liamputtong Pranee (2019). Handbook of Research Methods in Health Social Sciences.

[R10] Breuer S., Braun M., Tigard D., Buyx A., Müller R. (2023). How Engineers’ Imaginaries of Healthcare Shape Design and User Engagement: A Case Study of a Robotics Initiative for Geriatric Healthcare AI Applications. ACM Transactions on Computer-Human Interaction.

[R12] Byrne David (2022). “A Worked Example of Braun and Clarke’s Approach to Reflexive Thematic Analysis.”. Quality & Quantity.

[R13] Cheon E., Su N. M. (2018). “Futuristic Autobiographies: Weaving Participant Narratives to Elicit Values around Robots.”.

[R14] Crabtree A (1998). “Ethnography in Participatory Design.”. http://iotdatabox.com/IoT/EP_M001636_1.htmlViewproject.

[R15] Crabtree A., Nichols D. M., O’Brien J., Rouncefield M., Twidale M. B. (2000). “Ethnomethodologically Informed Ethnography and Information System Design.”. *Journal of the American Society for Information Science*.

[R16] Cumbo Bronwyn. n (2019). “Child Participation beyond the Adult Realm: Participatory Design in Nature-Play Contexts”.

[R17] Cumbo B., Selwyn N. (2022). “Using Participatory Design Approaches in Educational Research.”. International Journal of Research & Method in Education.

[R19] Dindler C., Iversen O. S. (2007). “Fictional Inquiry—Design Collaboration in a Shared Narrative Space.”. CoDesign.

[R20] Drain A., Shekar A., Grigg N. (2021). “Insights, Solutions and Empowerment: A Framework for Evaluating Participatory Design.”. CoDesign.

[R21] Druin A (1999). “Cooperative Inquiry: Developing New Technologies for Children with Children.”.

[R22] Ehn P (2017). Participatory Design: Principles and Practices.

[R23] Forlano Laura (2017). “Posthumanism and Design.”. She Ji.

[R24] Frauenberger Christopher (2020). “Entanglement HCI The Next Wave?”. ACM Transactions on Computer-Human Interaction.

[R25] Galvão L. F. O., García L. S., Felipe T. A. (2021). “Distributed Participatory Design Platform for Game Design with Deaf Children Distributed Participatory Design Platform for Game Design with Deaf.”.

[R26] Guha M. L., Druin A., Fails J. A. (2013). “Cooperative Inquiry Revisited: Reflections of the Past and Guidelines for the Future of Intergenerational Co-Design.”. International Journal of Child-Computer Interaction.

[R27] Hall A., Wojdecka A., College R. (2006). De-Anthropocentrising Healthcare Design.

[R28] Hamidi F., Mbullo P., Onyango D., Hynie M., McGrath S., Baljko M. (2018). “Participatory design of DIY digital assistive technology in Western Kenya.”.

[R30] Hanson W. E., Creswell J. W., Clark V. L. P., Petska K. S., Creswell J. D. (2005). “Mixed Methods Research Designs in Counseling Psychology.”. Journal of Counseling Psychology.

[R31] Hardy J., Vargas S. (2019). “Participatory Design and the Future of Rural LGBTQ Communities.”.

[R32] Hussain Sofia (2010). “Empowering Marginalised Children in Developing Countries through Participatory Design Processes.”. *CoDesign*.

[R33] Iivari N., Sharma S., Ventä-Olkkonen L., Molin-Juustila T., Kuutti K., Holappa J., Kinnunen E. (2022). “Critical Agenda Driving Child–Computer Interaction Research—Taking a Stock of the Past and Envisioning the Future.”. International Journal of Child-Computer Interaction.

[R34] Iversen O. S., Smith R. C., Dindler C.

[R35] Jarke J., Gerhard U. (2018). “Using Probes for Sharing (Tacit) Knowing in Participatory Design: Facilitating Perspective Making and Perspective Taking.”. I-Com.

[R37] Jasanoff (2015). Dreamscapes of Modernity: Sociotechnical Imaginaries and the Fabrication of Power.

[R36] Jasanoff S (2004). “States of Knowledge: The Co-Production of Science and the Social Order”.

[R38] Johnson B. D (2011). “Science Fiction Prototyping: Designing the Future with Science Fiction.”. Synthesis Lectures on Computer Science.

[R39] Jussli A., Gewald H.

[R40] Kakoudaki D (2014). Anatomy of a Robot: Literature, Cinema, and the Cultural Work of Artificial People.

[R43] Lee H. R., Sabanovi S., Chang W. L., Nagata S., Piatt J., Bennett C., Hakken D. (2017). “Steps Toward Participatory Design of Social Robots: Mutual Learning with Older Adults with Depression.”.

[R45] Malinverni L., Mora-Guiard J., Padillo V., Mairena M. A., Hervás A., Pares N. (2014). “Participatory design strategies to enhance the creative contribution of children with special needs.”.

[R80] McNeil M., Arribas-Ayllon M., Haran J. (2016). The Handbook of Science and Technology Studies.

[R47] Miller R., Brewer J. (2015). The A-Z of Social Research.

[R41] Moline K (2021). “Changing the Rules of the Game: Data and Ethnographic Surrealism Keywords”.

[R49] Mumford E (2006). "The Story of Socio-Technical Design: Reflections on Its Successes, Failures and Potential".

[R50] Nägele L. V., Ryöppy M., Wilde D. (2018). “PDFI: Participatory Design Fiction with Vulnerable Users.”.

[R51] Newbutt N., Rice L., Lemaignan S., Daly J., Charisi V., Conley I. (2022). Interaction Studies. Social Behaviour and Communication in Biological and Artificial Systems.

[R52] Nygaard K., Bergo O. T. (1974). “The Trade Unions New Users of Research:,”.

[R53] Oliver H (2019). “Design Fiction for Real-World Connected Wearables.”.

[R54] Porayska-Pomsta K., Frauenberger C., Pain H., Rajendran G., Smith T., Menzies R., Foster M. E. (2012). “Developing Technology for Autism: An Interdisciplinary Approach.”. Personal and Ubiquitous Computing.

[R55] Ray Murray P., Bagalkot N. L., Srivatsa S., Anthony P. (2021). “Design Beku: Toward Decolonizing Design and Technology through Collaborative and Situated Care-in-Practices.”. Global Perspectives.

[R56] Read J. C., Fitton D., Horton M. (2014). “Giving Ideas an Equal Chance: Inclusion and Representation in Participatory Design with Children.”.

[R57] Read J. C., Horton M., Sim G., Gregory P., Fitton D., Cassidy B. (2013). “CHECk: A Tool to Inform and Encourage Ethical Practice in Participatory Design with Children.”.

[R58] Rhee J (2018). “The Robotic Imaginary: The Human and the Price of Dehumanized Labor.”.

[R59] Rice Louis (2018). “Nonhumans in Participatory Design.”. CoDesign.

[R60] Robertson T., Simonsen J. (2012). “Challenges and Opportunities in Contemporary Participatory Design.”. Design Issues.

[R61] Rose E. J., Björling E. A. (2017). “Designing for Engagement.,”.

[R62] Šabanović S (2010). “Robots in Society, Society in Robots: Mutual Shaping of Society and Technology as a Framework for Social Robot Design.”. International Journal of Social Robotics.

[R64] Šabanović S., Chang W. L., Bennett C. C., Piatt J. A., Hakken D. (2015). “A Robot of My Own: Participatory Design of Socially Assistive Robots for Independently Living Older Adults Diagnosed with Depression.”.

[R65] Sanders E. B.-N (2000). “Generative Tools for Co-Designing.”. Collaborative Design.

[R66] Seale J., Colwell C., Coughlan T., Heiman T., Kaspi-Tsahor D., Olenik-Shemesh D. (2021). “‘Dreaming in Colour’: Disabled Higher Education Students’ Perspectives on Improving Design Practices That Would Enable Them to Benefit from Their Use of Technologies.”. Education and Information Technologies.

[R67] Seppälä T., Sarantou M., Miettinen S. (2016). “Arts-Based Methods for Decolonising Participatory Research.”.

[R68] Shahin M., Hussain W., Nurwidyantoro A., Perera H., Shams R., Grundy J., Whittle J. (2021). “Operationalizing Human Values in Software Engineering: A Survey.”. http://arxiv.org/abs/2108.05624.

[R69] Simonsen J., Robertson T. (2012). Routledge International Handbook of Participatory Design.

[R70] Spiel K., Brulé E., Frauenberger C., Bailley G., Fitzpatrick G. (2020). “In the Details: The Micro-Ethics of Negotiations and in-Situ Judgements in Participatory Design with Marginalised Children.”. *CoDesign*.

[R71] Stegner L., Senft E., Mutlu B. (2023). “Situated Participatory Design: A Method for In Situ Design of Robotic Interaction with Older Adults”.

[R72] Stimson C. E., Roy K., Szollosy M. (2024). “The Use of Participatory Design in Robotics: A Scoping Review”. Co-Design.

[R73] Sundblad Y (2010). “UTOPIA: Participatory Design.”.

[R74] Taylor Charles (2002). “Modern Social Imaginaries.”. Public Culture.

[R75] Wakkary Ron (2020). “Nomadic Practices: A Posthuman Theory for Knowing Design.”. International Journal of Design.

[R76] Wheeler G., Mills N., King S., Culmer P. (2018). “Child-Led, Creative Exploration of Paediatric Incontinence:”.

[R77] Wilson D. H (2015). “Chasing Our Science Fiction Future.”.

[R78] Winkle K., Senft E., Lemaignan S. (2021). “LEADOR: A Method for End-To-End Participatory Design of Autonomous Social Robots.”. Frontiers in Robotics and AI.

[R79] Yip J. C., Sobel K., Pitt C., Lee K. J., Chen S., Nasu K., Pina L. R. (2017). “Examining Adult-Child Interactions in Intergenerational Participatory Design.”.

